# The Landscape of Non-Viral Gene Augmentation Strategies for Inherited Retinal Diseases

**DOI:** 10.3390/ijms22052318

**Published:** 2021-02-26

**Authors:** Lyes Toualbi, Maria Toms, Mariya Moosajee

**Affiliations:** 1 UCL Institute of Ophthalmology, London EC1V 9EL, UK; l.toualbi@ucl.ac.uk (L.T.); maria.toms.14@ucl.ac.uk (M.T.); 2 The Francis Crick Institute, London NW1 1AT, UK; 3 Moorfields Eye Hospital NHS Foundation Trust, London EC1V 2PD, UK; 4 Great Ormond Street Hospital for Children NHS Found Trust, London WC1N 3JH, UK

**Keywords:** inherited retinal disease, non-viral gene therapy, plasmid DNA, nanoparticles, transfection, photoreceptors, retinal pigment epithelium

## Abstract

Inherited retinal diseases (IRDs) are a heterogeneous group of disorders causing progressive loss of vision, affecting approximately one in 1000 people worldwide. Gene augmentation therapy, which typically involves using adeno-associated viral vectors for delivery of healthy gene copies to affected tissues, has shown great promise as a strategy for the treatment of IRDs. However, the use of viruses is associated with several limitations, including harmful immune responses, genome integration, and limited gene carrying capacity. Here, we review the advances in non-viral gene augmentation strategies, such as the use of plasmids with minimal bacterial backbones and scaffold/matrix attachment region (S/MAR) sequences, that have the capability to overcome these weaknesses by accommodating genes of any size and maintaining episomal transgene expression with a lower risk of eliciting an immune response. Low retinal transfection rates remain a limitation, but various strategies, including coupling the DNA with different types of chemical vehicles (nanoparticles) and the use of electrical methods such as iontophoresis and electrotransfection to aid cell entry, have shown promise in preclinical studies. Non-viral gene therapy may offer a safer and effective option for future treatment of IRDs.

## 1. Introduction

Inherited retinal diseases (IRDs) are a group of genetically and phenotypically heterogeneous conditions affecting around one in 1000 people worldwide, with degeneration of the retina resulting in progressive loss of vision [1]. Extensive progress has been made towards understanding the pathophysiology of IRDs through advances in molecular genetic testing with at least 300 different causative genes identified (RetNet; https://sph.uth.edu/retnet/ (accessed on 1 February 2021)). So far, among the innovative therapeutic options, gene augmentation therapy has shown great promise by delivering healthy copies of the defective gene to the target tissue. The eye is an advantageous organ for such interventions due to its immune-privilege status and small enclosed structure allowing the use of a small amount of the vectors. In the case of targeting photoreceptor cells or the retinal pigment epithelium (RPE), subretinal injection allows the vectors to be directly delivered to the location of the target cells with no epithelial barriers or anatomical barriers. In contrast, intravitreal injection requires the vectors to bypass the inner limiting membrane and several subsequent cell layers before it reaches the photoreceptors and RPE. The development of ocular gene therapy benefits from the existence of numerous relevant animal models for preclinical investigations. In addition, many IRDs have a relatively slow progression and only become severe at later stages of life, providing a large therapeutic window. Furthermore, non-invasive in vivo imaging techniques allow reliable monitoring of the natural history of disease, and for measuring treatment safety and efficacy in clinical trials. Altogether, these features place the retina at the forefront of translational gene therapy.

Currently, there are over 40 gene therapy clinical trials for IRDs, ranging from phases I to III. Furthermore, the first viral gene therapy (Luxturna; voretigene neparvovec) gained FDA approval in 2018 for treating Leber congenital amaurosis (LCA) type 2, caused by mutations in *RPE65* [2]. While several systems have been developed and optimized to carry transgenes, the most efficient method remains the use of viral vectors, particularly adeno-associated virus (AAV) vectors. However, several challenges remain to be addressed, such as expanding the vector carrying capacity, ensuring long-term transgene expression, preventing genome integration, and keeping harmful inflammatory reactions to a minimum. Therefore, the development of alternative vectors is crucial to broaden the gene therapy options for IRDs.

Non-viral gene delivery systems are typically composed of the required nucleic acid (e.g., plasmid) complexed with a vehicle, such as cationic lipids or polymers, to aid cell entry. These vectors are cost-effective to manufacture, have potentially high carrying capacities, and may allow repeat administrations due to their safety profile. There has been a growing interest in non-viral gene therapies over the years, with a number of clinical trials completed and ongoing to treat a range of diseases, including cystic fibrosis and several types of cancer [3]. Although they may address the disadvantages of viral vectors, non-viral methods have not demonstrated transfection efficiencies in the retina comparable to AAV, and a number of chemical and physical-based strategies are under development to overcome this limitation. This review will describe the range of approaches for non-viral gene augmentation therapy that have been investigated for IRD treatment in the literature.

## 2. DNA Vector Design

**Figure 1:**
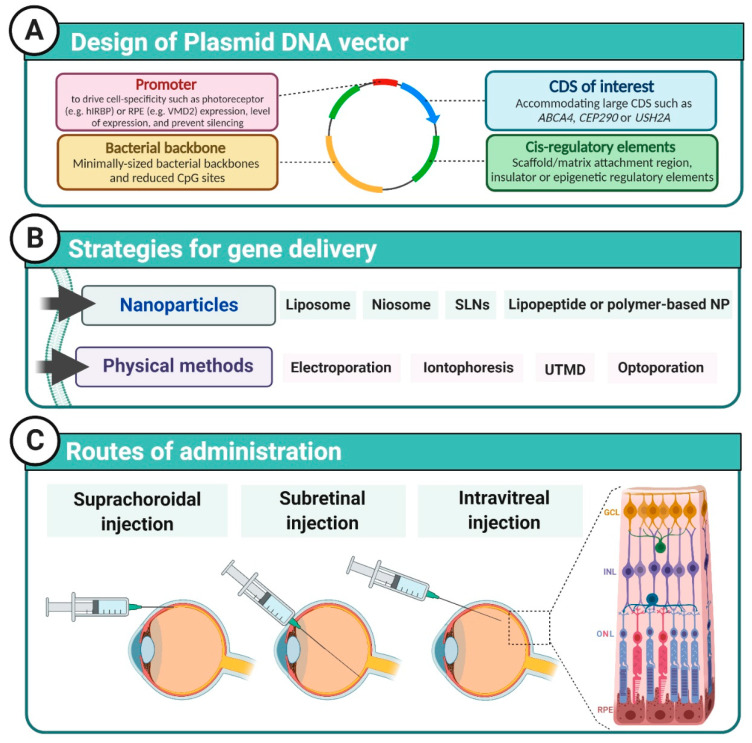
Retinal non-viral gene therapy. (**A**) Plasmid DNA vectors are designed to ensure cell-specific, long-lasting, and safe expression of the transgene of interest. (**B**) Different physical or chemical strategies can be applied to aid DNA transfection in the necessary cells (**C**) Different routes of administration can be used depending on the gene delivery strategy and the targeted cells. CDS: coding sequence; GCL: ganglion cell layer; INL: inner nuclear layer; ONL: outer nuclear layer; SLNs: solid-lipid nanoparticles; UTMD: ultrasound-targeted microbubble destruction (Created with BioRender.com (accessed on 1 February 2021)).

The use of plasmid-based DNA is the conventional approach for non-viral gene therapy. A plasmid vector consists of circular double-stranded DNA, typically including a promoter sequence, the coding sequence of the gene of interest, and regulatory sequences such as poly-adenylation sequences (Figure 1). Plasmid DNA is a fundamental tool for non-viral therapeutic strategies, as it influences (1) the transfection efficiency, (2) plays a key role in the transgene level and duration of expression, and (3) drives cell-specific expression. Although they need a combined delivery method, plasmid vectors have many advantages compared to viral vectors; they are cheap and easy to produce and to store, versatile, have a high cloning capacity, and display lower risks of integration and immunogenicity. While the AAV carrying capacity does not exceed 5 kb, plasmid vectors can virtually accommodate any gene insert size, making them suitable for larger IRD-associated genes such as *ABCA4*, *CEP290,* or *USH2A*. However, the larger the plasmid, the more toxic and inefficient the transfection [4,5]. Plasmid toxicity is correlated to size but also to unmethylated cytosine-phosphate-guanine dinucleotide (CpG) motifs, which are enriched in bacterial backbones [6]. Horizontal transfer of antibiotic resistant genes from plasmid DNA to normal microbial flora is also a safety concern. Consequently, much effort has been made to design plasmids free of antibiotic resistance cassettes and origin of replication (*ori*) with a minimal-sized or absent bacterial backbone in order to increase the transfection efficiency and decrease transgene silencing [7].

Promoters are the key players of transgene expression levels and cell specificity, and therefore optimized promoters are crucial for IRD gene therapy [8]. This issue is common to both viral and non-viral approaches in gene therapy. For instance, in the case of optogenetic therapies targeting the cone photoreceptors, it is important to choose the right AAV capsids and cell-type specific promoter combination for expression in cones, preventing any ectopic expression in other retinal or non-retinal cell types that would potentially lead to lower efficacy and increased toxicity [9,10]. Similarly, rhodopsin overexpression driven by a cytomegalovirus (CMV) immediate-early promoter in the wild-type mouse retina resulted in retinal toxicity [11]. Overall, transgene product overexpression or ectopic expression can lead to cellular stress. Khabou and collaborators reported that AAV vectors encoding green fluorescent protein (GFP) were significantly more toxic compared to a non-coding control capsid in mice retinas. Intracellular pathways such as endoplasmic reticulum (ER) stress can lead to apoptosis of transfected cells, and therefore reduce efficiency of the treatment [9]. Additionally, some promoters may be more prone to methylation and silencing [12], affecting the levels of the transgene expression and its maintenance with time. Therefore, a subtle balance between transgene expression and functional rescue must be found to ensure an efficient and safe gene therapy.

Further improvements of plasmid DNA constructs have been explored, notably with the addition of anti-repressor elements or epigenetic regulators. Among them, scaffold matrix attachment region (S/MAR)-containing DNA vectors have shown promising results as a non-viral gene therapy strategy, especially for treatment of RPE-based diseases. S/MARs are sequences found in eukaryotic genomes that anchor the chromatin to the nuclear matrix [13]. They are 300 to 3000 bp-long with 70% AT-rich content [14]. The first report of these motifs was published more than 30 years ago in a study of Drosophila DNA, where they were suspected to play a role in chromatin loop organization [15]. Subsequently, several S/MAR sequences have been characterized in the human genome, such as the *APOB* MAR associated with the human apolipoprotein B locus, or the *IFNB1* MAR associated with the human Interferon Beta 1 locus [16]. S/MAR elements that occur in the genome are thought to contribute to DNA structure, loop domain partitions, replication and transcriptional activity regulation [17]. The AT-rich content of the S/MAR sequence has been shown to favor DNA strands unwinding, increasing its availability to transcriptional factors [18]. Additionally, S/MAR sequences have been found to play an insulator-like role by protecting genes from being silenced [19], and they serve as binding sites for scaffold attachment factor protein A (SAF-A), an RNA-binding protein that interacts with p300 [20]. The recruitment of several other transcription activators is facilitated by S/MAR sequences such as SAF-B, SATB1, and ARBP [21,22].

The first virus-free episomal vector exploiting the valuable properties of S/MAR sequences was the pEPI vector, which was capable of replicating in eukaryotic cells (Chinese hamster ovary [CHO] cells). The cloned 2 kb S/MAR fragment in this vector came from the 5′ region of the human interferon β-gene and was responsible for stable gene expression maintained over more than 100 generations without selection pressure [23]. Following this work, the development of a minimally-sized S/MAR vector, produced by cutting off the bacterial backbone of pEPI, allowed a higher and more sustained expression both in vitro and in vivo [24]. This pEPI minicircle contains the expression cassette of interest and a 2 kb S/MAR fragment. Minimally-sized plasmid vectors improve the efficiency not only by reducing the plasmid size, but also by removing bacterial CpG motifs that can result in innate immune responses and vector silencing [25].

Subsequently, many improvements have been made, such as the addition of insulating elements and the production of spliced versions of the vectors with a minimal bacterial backbone to increase vector expression and establishment [25]. These DNA vectors present several advantages for gene therapy such as (1) persistent expression and episomal maintenance without insertional mutagenesis, (2) high cloning capacity that can accommodate the cDNA of large genes, (3) no potentially toxic viral components, and (4) great versatility and accessible production.

For retinal treatment, Koirala and colleagues developed a promising non-viral approach, utilizing S/MAR DNA vectors in a LCA mouse model. VMD2-hRPE65-S/MAR plasmid nanoparticles were introduced into *Rpe65*^−/−^ mice by subretinal injection. In mice at 15 months post-injection, expression of human RPE65 protein in the RPE, functional rescue of the protein, and improvement of cone electroretinogram (ERG) amplitudes were detected [26,27]. Similarly, another study assessed the sustained expression of several S/MAR vectors such as pEPI and its derivative pEPI in the mouse retina and demonstrated transgene expression up to 32 days post-injection [28]. More recently, the addition of non-coding components of genomic DNA from the rhodopsin gene into a plasmid vector resulted in eight months of functional and structural improvements in a rhodopsin knockout mouse model [29,30,31]. Ultimately, improvements in plasmid DNA vectors, such as more efficient and relevant promoters, the use of cis-regulatory elements, and minimally sized plasmids are paving the way for better non-viral ocular gene therapy.

## 3. Nanoparticles

**Figure 2:**
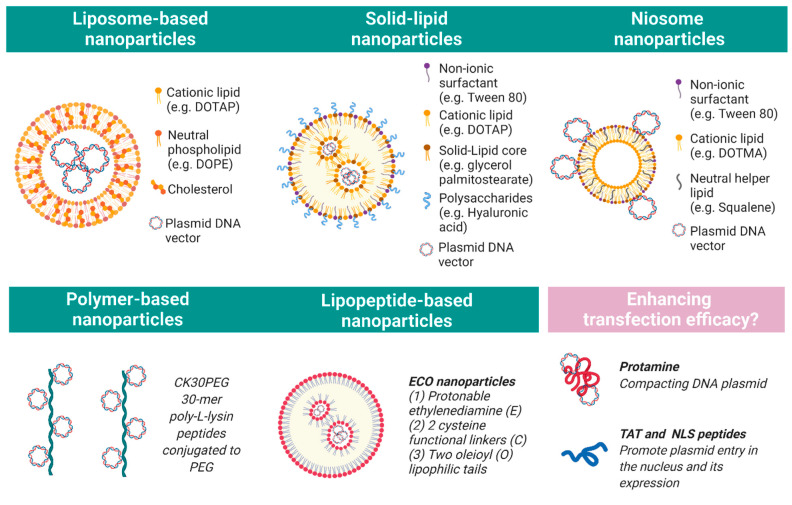
Key nanoparticles of interest for retinal non-viral gene therapy. Several chemical vehicles have been developed as plasmid DNA carriers, such as nanoparticles based on liposomes, solid lipids, niosomes, polymers, and lipopeptide (Created with BioRender.com (accessed on 1 February 2021)).

The efficiency of naked DNA transfection is very low, and therefore several synthetic carriers have been developed to use in combination with nucleic acids to assist cell entry (Figure 2). Nanoparticles are cationic structures capable of forming a complex with polyanionic nucleic acids. This complex facilitates cell uptake from the endosomal cellular system to the nucleus and protects the transgene from endonucleases. This section will review the main proof-of-concept studies in IRD animal models using synthetic vectors based on liposomes, polymers, solid lipids, and niosomes (Table 1).

### 3.1. Liposomes

Liposomes are nanoparticles made of a phospholipid bilayer allowing molecule encapsulation. For transfection of the retina, Rajala and colleagues developed an innovative liposome-based protamine complex with improved efficiency and long-term expression. Their next-generation lipoplex contained (1) a liposome consisting of a cationic lipid DOTAP (1, 2-dioleoyl-3- trimethylammonium-propane), a neutral lipid DOPE (1, 2-dioleoyl-sn-glycero-3-phosphoethanolamine), and cholesterol; (2) protamine to compact the DNA and protect it from endonucleases; and (3) cell penetrating transactivator of transcription (TAT) and nuclear localization signaling (NLS) peptides to promote plasmid entry in the nucleus and its expression. Subretinal injection of this liposome-based nanocarrier, coupled with an *Rpe65* DNA plasmid, successfully resulted in the efficient transfection of photoreceptor and RPE cells of an *Rpe65^−/−^* mouse, partially rescuing the disease phenotype. GFP expression was reported at three months post-injection [32]. Following this work, Wang and colleagues performed liposome-based retinal transfections using cell-specific promoters; they achieved specific gene expression in the RPE with the VMD2 promoter, ganglion cells with the thymocyte antigen promoter, and finally rod and cone photoreceptors with the mouse rhodopsin and red opsin promoters, respectively [39]. Overall, these liposome-based complexes provide a promising alternative to viral vectors for retinal gene therapy, but assessment in larger animal models is necessary.

### 3.2. Polymers

Among the polymer nanoparticle formulations investigated for retinal gene therapy, poly-l-lysin peptides have shown convincing results. Naash’s group have used compacted rod-shaped DNA nanoparticles formulated with 30-mer poly-l-lysin peptides conjugated to polyethylene glycol 10,000K (CK30PEG) in a number of investigations; they have successfully shown efficient transfection of photoreceptors and RPE cells, which improved the phenotype of several retinal mouse disease models such as retinitis pigmentosa [35] and Leber congenital amaurosis, with up to two years of persistent transgene expression [27]. In another study, CK30PEG nanoparticles were enhanced with a cell penetrating TAT peptide sequence and demonstrated partial improvement of visual function in the *Rho^P23H/P23H^* knock-in mouse model of retinitis pigmentosa [31]. Furthermore, CK30PEG containing the large *ABCA4* cDNA cassette (6.8 kb) was able to drive sustained expression for up to eight months after injection and to improve the phenotype of an *Abca4*-deficient Stargardt disease mouse model when delivered subretinally [36]. Plasmids as large as 20 kb have been effectively transfected using CK30PEG for in vivo mice lung gene transfer [40], which is promising for large transgene delivery in the eye. Encouragingly, translation in a non-human primate eye showed safe and efficient transfection of CK30PEG when injected subretinally and intravitreally [41]. In addition, anionic span-based poly-l-arginine nanoparticles have been used to deliver a *PRFP31* plasmid and partially rescue the retinal phenotype in *Prpf31^A216P/+^* mouse model of retinitis pigmentosa [42].

More recently, a combined strategy using a lipo-peptide nanoparticle showed efficient plasmid DNA delivery into retinal cells. These so-called ECO nanoparticles consist of a protonable ethylenediamine (E) head group, two cysteine (C) functional linkers, and two oleoyl (O) lipophilic tails [43]. In the eye, these nanoparticles, self-assembled by the multifunctional pH-sensitive amino lipid ECO and a therapeutic bovine rhodopsin promoter-driven *ABCA4* plasmid, delayed the phenotype of an *Abca4^−/−^* Stargardt mouse model for at least six months [44]. The 16 kb plasmid is the largest reported for non-viral gene therapy in the eye. Prolonged ABCA4 expression for at least eight months was observed in the photoreceptor outer segments of subretinally injected mice. ECO-based nanoparticles can also be chemically modified with targeting ligands; the addition of a retinylamide or its analogue ACU4429 produced increased RPE expression in *Rpe65^−/−^* LCA model mice and *Abca4^−/−^* mice, respectively [37,38].

### 3.3. Chitosans

Alternative biopolymers such as chitosan have shown valuable properties as nanoparticle building blocks. Chitosan is a biodegradable non-toxic cationic polysaccharide. It is produced by alkaline deacetylation of chitin, which is a component commonly found in the cell walls of fungi and crustacean shells. Chitosans are composed of *N*-acetyl-d-glucosamine and d-glucosamine units and vary in molecular weight (50 to 2000 kDa) and in the degree of deacetylation (40–99%). The cationic nature of chitosan derivatives is an exception among the usual polysaccharides, which makes it an invaluable polymer as a non-viral gene vector component. Regarding ocular gene therapy applications, Puras and colleagues assessed the efficiency of highly deacetylated (99%) low molecular weight (5.7 kDa) oligochitosan-DNA nanoparticles in the rat retina. Subretinal injection led to GFP expression in the RPE cells, while intravitreal injection induced GFP expression in the retinal ganglion cells [45]. Similarly, Mitra and colleagues designed a chitosan-based nanoparticle (250 kDa, 82% of deacetylation degree) with glycol moieties to improve its solubility. Subretinal injections of 5.7 kb-long GFP plasmid DNA glycol chitosan nanoparticles in albino wild-type mice resulted in GFP expression in the RPE cells, without any safety concerns [46]. The safe profile of chitosan-derived nanoparticles makes them a strategy of interest for retinal gene therapy; however, modification and optimization still need to be explored to improve the low efficiency [47,48].

### 3.4. Solid Lipids

Similarly, solid lipid nanoparticles (SLNs) have displayed promising results as vectors for gene delivery. SLNs are 10–1000 nm-diameter nanocarriers with a rigid core lipid matrix [49]. They offer many advantages compared to liposomes and polymeric nanoparticles such as (1) their biodegradability, (2) their stability and large-scale production feasibility, and (3) the possibility of ligand additions [50]. Apaolaza and colleagues designed a solid lipid-based formulation consisting of DOTAP, protamine, and a polysaccharidic ligand such as hyaluronic acid or dextran. The protamine is a cationic peptide with nuclear localization signals enhancing DNA condensation [51], while hyaluronic acid contributes to better plasmid DNA cell delivery and the final structure of the SLNs [52]. SLNs coupled with an *RS1* plasmid driven by the murine opsin promoter (mOPS) successfully induced long-lasting photoreceptor-specific expression of RS1 (three months) in an X-linked juvenile retinoschisis mouse model when injected intravitreally, resulting in an improved phenotype [33,34]. The SLNs capability to reach the photoreceptors and RPE when injected intravitreally makes these nanoparticles a very promising feature for retinal gene therapy.

### 3.5. Niosomes

Niosome-based nanoparticles are similar to liposomes, except the phospholipid is replaced by non-ionic surfactants (reviewed in [53]). Niosomes are usually composed of three key elements: a non-ionic surfactant as its main component, a cationic lipid interacting with the plasmid DNA, and a neutral lipid helper. In the eye, several studies have been conducted using various niosome-based plasmid DNA carriers; initially, a cationic niosome formulation with 2,3-di(tetradecyloxy)propan-1-amine cationic lipid, combined with 2% of squalene and 0.5% of polysorbate 80, was optimized for compact delivery of a 5 kb-long pCMS-eGFP DNA plasmid [54]. Following subretinal injection in rats, RPE cells were modestly transfected, while intravitreal injection led to GFP expression in the inner retinal layers. The addition of protamine to the formulation improved nucleus targeting and allowed transfection of a small proportion of photoreceptor cells following subretinal injection, although the transfection efficiency remained very modest. Several combinations have been assessed by changing the non-ionic surfactant [55], the lipid helper [56], or the cationic lipid [57].

## 4. Physical Methods of Transfection

To facilitate cell entry of non-viral gene therapies, several physical methods have been developed to allow plasmids to efficiently cross cell barriers and be expressed. Among those reported to increase transfection in retinal cells are iontophoresis, electrotransfection, ultrasound-targeted microbubble destruction (UTMD), and optoporation.

Iontophoresis is a non-invasive drug delivery technology enhancing the permeation of ionized molecules across biological barriers using a continuous low-level electrical field [58]. This strategy has been proven useful for transdermal drug delivery to facilitate percutaneous penetration [59,60] and became an attractive option for drug and gene delivery in the eye [61]. Several studies have assessed transcorneal and transscleral iontophoresis-assisted plasmid DNA transfer for non-viral ocular gene therapy; however, limited expression was produced, especially in the photoreceptor cells [58,62]. Asahara and colleagues applied iontophoresis to transfect a 4.7 kb-long CMV-GFP plasmid in rabbit eyes and showed expression in the cornea, the anterior chamber angle, and the ciliary subepithelial tissues, but not in the retina [62]. In contrast, Souied and colleagues demonstrated that trans-scleral iontophoresis of *β-pde6b* cDNA plasmid driven by the human PDE6B promoter in *rd1* mice could penetrate photoreceptor cells and consequently showed partial rescue of photoreceptor morphology and ERG measurements [58]. For both studies, the positive electrode was placed at the back of the animal, while the negative electrode was inserted into an applicator containing the plasmid solution bathing the cornea, the limbus, and the adjacent sclera. Overall, the safety profile and non-invasive aspects of iontophoresis are ideal for non-viral retinal gene delivery strategies and would allow safe repetitive applications, such as those performed in *rd1* mice by Souied et al. [58]. However, no novel studies using this technique have been reported in the last decade, and it would still require extensive optimization regarding its low plasmid transfection in retinal cells. This technology appears to be most suitable for small molecules and short nucleic acids [63].

Electrotransfection, also known as electroporation, is an additional method exploiting electric fields that has been explored for non-viral gene delivery. Unlike iontophoresis, electrotransfection relies on a high voltage pulsed electric field applied to the surrounding cells, which transiently permeabilizes their cell membranes, allowing plasmid DNA entry. In the retina, successful plasmid electrotransfection following a subretinal injection has been performed in newborn mouse and rat (P0) retinal cells [64,65,66,67] and in adult mouse RPE cells [68,69]. Alternative routes of delivery, such as injection of plasmid DNA solution into the suprachoroidal space followed by electrotransfection, displayed transfection of choroid, RPE, and a proportion of photoreceptor cells in the adult rat [70]. Altogether, these studies demonstrate the valuable features of electrotransfection such as (1) its efficiency of transfection, (2) the possibility for repetitive administrations, and (3) its cost effectiveness compared to viral vectors. However, the therapeutic use of electrotransfection would require invasive surgical procedures and extensive optimization to ensure safety in patients. In addition, the application of a safe and adapted electric field to the large 1094 mm^2^ human retinal surface is a challenge that will need to be addressed. To date, no proof-of-concept investigations in large animal eyes have been reported. Retinal electrotransfection would benefit from alternative approaches using innovative electrotransfection tools to address these issues [71].

Other physical methods under investigation include ultrasound-targeted microbubble destruction (UTMD). This involves loading plasmid DNA into microbubbles, which are small gas-filled spherical voids stabilized with phospholipids or synthetic polymers. Gene-carrier microbubbles are injected and subjected to ultrasounds; the microbubbles act as cavitation nuclei by focusing ultrasound energy, causing cell membrane permeabilization and plasmid uptake [72]. UTMD is non-invasive, allows repetitive administrations, and displays low toxicity, but with limited transfection in RPE and photoreceptor cells [73,74].

More recently, optoporation has been investigated as a method of gene transfer in the retina. Laser-induced optoporation allows the introduction of small molecules or plasmid DNA by transiently permeabilizing cell membranes using continuous or pulsed laser waves [75]. Batabyal and colleagues have successfully used this strategy to efficiently deliver a 7.9 kb-long plasmid in *rd10* mouse retinal ganglion cells. They designed a two-step strategy: the first step is an intravitreal injection of the plasmid of interest and gold nanorods conjugated with concanavalin A to the target cell membrane; the following step is an 800–1064 nm laser irradiation, allowing site-specific cell permeabilization [76,77]. Additionally, no evidence of a harmful immune response or other safety issues was apparent, making optoporation-based gene delivery a promising tool for retinal gene therapy.

Overall, a number of physical methods have shown great potential as delivery strategies for non-viral gene therapy, but improving their transfection efficiency and translation to larger animal models is essential. Several of these techniques can also be used as an adjuvant method to increase viral vectors penetrance or transduction efficiency. For instance, intravitreal injection of AAV vectors combined with iontophoresis significantly improved penetration of the internal limiting membrane and increased transduction of cells in the outer retina [78,79]. Similarly, several studies showed that UTMD-mediated delivery of AAV improved their transduction in rodent retinas in vivo [73,80].

## 5. Limitations of Non-Viral Ocular Gene Therapy

The main limitation of non-viral strategies for gene augmentation therapy is their lack of transfection efficiency in targeting the photoreceptor cells and RPE. So far, non-viral strategies still do not outperform the AAV transduction rates. Han and colleagues performed a comparative analysis of CK30PEG, AAV2, and AAV5 efficacies with subretinal injections in mice [81]. They showed that the AAV vectors (10^9^ vg) were more efficient per vector genome than CK30PEG (6.9^11^ vg), but CK30PEG still drove a comparable level and longevity of gene expression. However, AAV capsids have been subsequently improved, such as the engineered AAV2.7m8 capsid [82], allowing highly efficient transduction after both subretinal and intravitreal injection.

In the case of AAV vector-based gene therapies, several clinical trials have reported cases of inflammation in treated eyes. For instance, an inflammatory response to a high dose of AAV2-*RPE65* was reported in five of eight participants, displaying intraocular inflammation, which was resolved under steroid treatments [83]. In addition, one serious adverse event of presumed intra-retinal inflammation resulting in severe functional and structural impairment was observed in a clinical trial treating choroideremia patients with AAV2-*REP1* [84]. Furthermore, preclinical studies in non-human primates reported an innate and adaptative immune response following subretinal injection of clinical-grade AAV8 under concomitant steroid treatment [85]. This activated all three main recognition pathways of innate immunity: toll-like, NOD-like, and RIG-I-like [85]. These receptors play the role of microbial sensors to bacterial or viral products and nucleic acids and mediate the innate immune response. Some clinical trials use prophylactic and/or perioperative steroid administration to prevent or limit inflammatory responses in the eye [86,87]. More recently, Chan and colleagues engineered AAV incorporating short DNA oligonucleotides antagonizing TLR9 activation, which reduced innate immune and T cell responses and enhanced transgene expression [88].

Although non-viral methods are less likely to trigger significant inflammatory responses because they lack the viral capsid, the potential responses still require investigation. For instance, double-stranded DNA can trigger an innate immune response mediated by the toll-like receptor pathway or cGAS pathway [89]. Furthermore, physical methods of transfection can lead to inflammation through damage-associated molecular patterns (DAMPs), by releasing intracellular proteins, extracellular matrix, or non-protein molecules like ATP [90]. Altogether, these safety issues need to be assessed in a relevant retinal context. Similar to AAV therapy, steroid administration may be necessary alongside the non-viral gene therapy to reduce potential inflammatory responses.

Investigation into non-viral methods for retinal gene therapy should be intensified to overcome its current limitations and reach the clinical stage. To date, only one study assessed DNA nanoparticles in non-human primate eyes [41]. No adverse events were reported in the injected baboons, with no systemic or inflammatory reaction subsequent to the injections. However, the efficiency was not as high as current AAV capsids used in clinical trials. Similarly, none of the non-viral physical transfection methods have reached the clinical stage for IRDs. However, the Evesensys electroporation system is currently being trialed for electroporating the eye ciliary muscle in patients with uveitis, allowing transfected cells to produce and secrete therapeutic proteins of interest (NCT03308045). The company is aiming to use the same technology to treat patients with degenerative retinal diseases. 

## 6. Conclusions

The development of gene augmentation therapy has created a highly promising avenue for treating a range of IRDs, which will greatly impact the quality of life of affected patients. With the first approved ocular gene therapy and a number of ongoing clinical trials, the development of enhanced strategies is of interest more than ever. Non-viral vectors offer a relatively cost-effective and safe alternative option, with greater packaging capacities compared to viral vectors. Currently, the common limitation for non-viral IRD treatment is the low transfection efficiency in the key retinal cells of interest, the photoreceptors. There are various aspects of the non-viral therapeutic strategy that can be targeted for optimization, including DNA plasmid design, chemical delivery vehicles, and injection techniques. Innovative improvements, and assessing different gene transfer methods in combination, will be necessary to ensure a sufficient transfection efficiency in the retina, with safe long-term expression. Further non-viral therapeutic studies in larger animal models will also aid clinical translation.

## Figures and Tables

**Table 1:** Non-viral retinal gene therapy studies in IRD animal models.

Nanoparticle Types	Gene	Plasmid DNA	Proof-of-Concept in IRD Animal Models	Reference
Liposome	*RPE65*	Promoter. CMV cDNA. *hRPE65*	Improved phenotype in *Rs1h*-deficient mouse model of XLRS	Rajala et al., 2014 [32]
SLNs	*RS1*	Promoter. CMV or mOPS cDNA. *RS1*	Partial phenotype rescue in *Rpe65*^−/−^ mouse model of LCA	Apaolaza et al., 2015, 2016 [33,34]
Polymer-based CK30PEG	*Rds*	Promoter. CMV or mOPS cDNA. *RS1*	Improved phenotype in *rds^+/−^* mouse model of RP	Cai et al., 2010 [35]
Polymer-based CK30PEG	*ABCA4*	Promoter. IRBP or Mops cDNA. *ABCA4*	Improved phenotype in *Abca4*^−/−^ mouse model of Stardgadt disease	Han et al., 2012 [36]
Polymer-basedCK30PEG	*RPE65*	Promoter. VMD2 cDNA. *hRPE65*	Improved phenotype rescue in *Rpe65*^−/−^ mouse model of LCA	Koirala et al., 2013 [27]
ECO nanoparticle	*ABCA4*	Promoter. Rho cDNA. *ABCA4*	Improved phenotype in *Abca4*^−/−^ mouse model of Stargardt disease	Sun et al., 2019 [37]
ECO nanoparticle	*RPE65*	Promoter. Not mentioned cDNA. *hRPE65*	Improved phenotype rescue in *Rpe65*^−/−^ mouse model of LCA	Sun et al., 2017 [38]

SLNs, Solid-lipid nanoparticles; CK30PEG, 30-mer poly-l-lysin peptides conjugated to polyethylene glycol 10000K; ECO, protonable ethylenediamine (E) head group, two cysteine (C) functional linkers, and two oleoyl (O) lipophilic tails.
